# Supplementary material for: Enhancing the emergency department experience for older adults: Study protocol for the implementation of a comfort menu and cart
Source: PLoS One. 2025 Dec 4;20(12):e0332773. doi: 10.1371/journal.pone.0332773 (PMC12677483; doi:10.1371/journal.pone.0332773)
Supplement: S1 File — (PDF) [file pone.0332773.s002.pdf]

PARECER CONSUBSTANCIADO DO CEP

DADOS DO PROJETO DE PESQUISA

Título da Pesquisa: Intervenções não farmacológicas para melhorar experiência do paciente idoso no pronto atendimento: implementação de menu e carro de conforto em um hospital filantrópico terciário

Pesquisador: Pedro Kallas Curiati

Área Temática:

Versão: 2

CAAE: 85870125.4.0000.5461

Instituição Proponente: Sociedade Beneficente de Senhoras Hospital Sírio-Libanês

Patrocinador Principal: Sociedade Beneficente de Senhoras Hospital Sírio-Libanês

DADOS DO PARECER

Número do Parecer: 7.436.261

Apresentação do Projeto:

Desenho:

Este estudo utilizará um desenho quase-experimental, do tipo antes e depois.

Resumo:

INTRODUÇÃO: O envelhecimento populacional é uma tendência global, com projeções indicando um aumento significativo na proporção de indivíduos com 65 anos ou mais até 2050. Esse cenário impõe a necessidade de adaptar os serviços de pronto atendimento (PA) para atender as demandas específicas dos pacientes idosos, que frequentemente apresentam múltiplas comorbidades e enfrentam desafios como dificuldades sensoriais e cognitivas. Diretrizes internacionais, como as publicadas pelo American College of Emergency Physicians e pela European Task Force on Geriatric Emergency Medicine, recomendam modificações no ambiente físico e no treinamento das equipes para melhorar o cuidado em PAs geriátricos. Em estudo recente, publicado em 2021, Lichen e cols. demonstraram que intervenções não-farmacológicas, como um "carro de conforto", melhoram a experiência de pacientes idosos no PA. OBJETIVOS: Avaliar o impacto da implementação de menu e "carro de conforto" em experiência de pacientes idosos atendidos no PA., experiência de profissionais das equipes médica e multidisciplinar envolvidos no cuidado de idosos no PA, tempo de permanência

hospitalar, custos hospitalares e outros desfechos centrados no paciente em até 30 dias, incluindo revisitas hospitalares, quedas, incidência de delirium, qualidade de vida, estado funcional e desempenho cognitivo. MÉTODOS: Será realizado um estudo de antes e depois em que serão recrutados pacientes maiores de 65 anos atendidos nos boxes privativos do PA do Hospital Sírio Libanês (HSL). Os critérios de inclusão para o estudo serão: atendimento no PA do HSL; idade igual ou maior a 65 anos; acomodação em box privativo, independentemente de indicação de internação; e capacidade de consentir e responder à entrevista ou presença de acompanhante habilitado a fazê -lo. Os critérios de exclusão incluirão: recusa em participar do estudo e/ou utilizar menu e carro de conforto; ausência de acompanhante capaz de consentir com a participação no estudo e fornecer as informações necessárias quando o paciente apresentar alteração do estado mental ou comprometimento cognitivo; rebaixamento do nível de consciência; instabilidade hemodinâmica; insuficiência respiratória aguda; e impossibilidade de contato telefônico para entrevista. Serão incluídos pelo menos 132 pacientes em cada fase do estudo (pré e pós implementação do carro de conforto). Os pacientes serão entrevistados inicialmente para obtenção de dados demográficos, clínicos e relacionados a sua experiência no PA. Na fase pós implementação do carro de conforto, serão adicionadas questões relativas à percepção sobre o impacto deste sobre experiência e conforto durante a permanência no PA. Entrevista telefônica de seguimento será realizada 30 dias após a inclusão para avaliação de desfechos clínicos de interesse, incluindo revisitas hospitalares, quedas, incidência de delirium, qualidade de vida, humor, funcionalidade, e desempenho cognitivo. As análises serão realizadas com o Stata 17, usando testes estatísticos bicaudais com erro alfa de 0,05. Variáveis numéricas serão apresentadas como médias e desvios padrão ou medianas e intervalos interquartis, conforme a distribuição. Variáveis categóricas serão descritas em contagens e proporções. Comparações de variáveis numéricas serão feitas com teste t de Student ou ANOVA para distribuições normais e teste de Wilcoxon ou Kruskal-Wallis para não normais. Variáveis categóricas serão comparadas com teste do qui-quadrado ou teste exato de Fisher, se necessário. RESULTADOS ESPERADOS: Esperamos demonstrar o impacto da implementação de menu e carro de conforto em PA em todos os desfechos estudados.

Introdução:
O envelhecimento da população é uma realidade incontestável tanto no Brasil quanto globalmente (1). De acordo com o relatório de 2019 do Departamento de Economia e Assuntos Sociais da Organização das Nações Unidas (ONU), a população mundial com 65 anos ou mais

aumentará de 9,1% para 11,7% em 2030 e 15,9% em 2050, superando o número de jovens com 15 a 24 anos (1). O número de pessoas com mais de 80 anos também está crescendo significativamente no mundo, com uma projeção de 426 milhões de indivíduos nessa faixa etária em 2050 (1).Pacientes idosos tendem a apresentar múltiplas comorbidades, o que os leva a procurar serviços de pronto atendimento (PA) com maior frequência (2, 3). No entanto, esses serviços frequentemente não estão adaptados para atender adequadamente as suas particularidades (2). Dificuldades visuais e auditivas, declínio na reserva funcional, comprometimento cognitivo devido a delirium e/ou demência, e comunicação prejudicada são problemas comuns que podem ser exacerbados pelo ambiente estressante do PA (2, 4).Para enfrentar esses desafios, diretrizes específicas de atendimento foram desenvolvidas por organizações internacionais. O American College of Emergency Physicians, em conjunto com a American Geriatrics Society, a Emergency Nurses Association, e a Society for Academic Emergency Medicine, publicou em 2014 o documento "Geriatric Emergency Department Guidelines" (4). Estas diretrizes contêm recomendações detalhadas sobre a modificação do ambiente físico, o treinamento de equipes e a implementação de processos de atendimento específicos para pacientes idosos (4). Estes incluem criação de áreas de espera mais confortáveis, uso de iluminação adequada, redução de ruído, e disponibilização de dispositivos de auxílio à marcha (4). Já a European Task Force on Geriatric Emergency Medicine publicou em 2020 recomendações para o atendimento de idosos no PA que enfatizam a importância de avaliação abrangente, incluindo triagem para condições comuns em idosos, como delirium, depressão e risco de quedas (5). Esse documento também sugere a implementação de protocolos específicos para o manejo de condições crônicas e agudas, bem como a integração de cuidados paliativos, quando apropriado (5). Uma recomendação comum a ambas as diretrizes é o treinamento contínuo da equipe de saúde em competências geriátricas, que incluem comunicação eficaz com pacientes idosos e identificação e manejo de síndromes geriátricas (4, 5). A designação de "geriatric champions" ou referências em geriatria dentro das equipes de emergência visa garantir que os cuidados sejam apropriados e baseados nas melhores práticas (4, 5).Um estudo realizado em PA nos Estados Unidos e publicado por Lichen e cols. em 2021 explorou o impacto de intervenções nãofarmacológicas na melhoria do conforto e da experiência de pacientes idosos (3). Esse estudo introduziu o conceito de "carro de conforto", um carrinho móvel contendo itens de baixo custo, como óculos de leitura, aparelhos auditivos, cobertores aquecidos, e materiais de entretenimento, como livros e revistas com letras grandes, com o objetivo de melhorar a comunicação entre os pacientes

idosos e a equipe de saúde, além de proporcionar uma experiência mais agradável durante a espera e o atendimento (3). O "carro de conforto" foi disponibilizado em áreas de espera e dentro das salas de atendimento, permitindo que os pacientes tivessem acesso a esses itens conforme necessário (3). Além disso, a equipe de enfermagem recebeu treinamento específico para utilizar esses recursos de forma eficaz e para identificar as necessidades individuais dos pacientes idosos. O impacto dessa intervenção foi avaliado através de questionários de satisfação preenchidos por pacientes e pela equipe de saúde antes e depois da implementação do carro de conforto, com resultados bastante positivos (3). Concluiu-se que intervenções simples, de baixo custo, humanizadas e centradas no paciente podem ter um impacto substancial na experiência dos pacientes e dos profissionais que os atendem no PA (3). De acordo com o nosso melhor conhecimento, não existem outros estudos sobre o uso de carro de conforto para idosos em ambientes de PA. Stolzman e cols., em 2020, exploraram uma intervenção análoga na unidade de terapia intensiva (UTI), liderada por enfermeiros, para melhorar o atendimento a pacientes críticos e suas famílias (6). A iniciativa englobou itens para acolhimento e enfatizou a importância de um ambiente que promovesse bem-estar, especialmente em situações de fim de vida (6). Este estudo também revelou o fortalecimento do trabalho em equipe e do cuidado centrado no paciente, com melhora da experiência na UTI e atenção às necessidades emocionais das famílias (6).McCusker e cols., em 2018, exploraram a experiência de pacientes idosos em PA e propuseram medidas para sua avaliação (2). Foram destacados os desafios específicos que esses pacientes enfrentam e indicado que suas necessidades podem diferir daquelas de pacientes mais jovens (2). Os autores criaram e validaram duas escalas para avaliação de aspectos relevantes dos cuidados em PA por idosos, uma direcionada para cuidados pessoais e comunicação e outra para tempo de espera, ambas significativamente associadas à percepção de qualidade do cuidado e a propensão a retornar para o mesmo serviço de PA (2). Propôs-se a sua aplicação para aprimorar a experiência dos idosos, adaptando o cuidado às suas necessidades específicas e promovendo um ambiente mais acolhedor e resolutivo para esse grupo (2).

Hipótese:
A implementação de um menu e um ¿carro de conforto¿ no PA do Hospital Sírio-Libanês (HSL) melhorará experiência de paciente e equipes assistenciais, assim como tempo de permanência hospitalar, custos hospitalares e outros desfechos centrados no paciente em até 30 dias, incluindo revisitas hospitalares, quedas, incidência de delirium, qualidade de vida,

funcionalidade, e desempenho cognitivo.

Metodologia Proposta:
Todos os participantes serão convidados a assinar termo de consentimento livre e esclarecido (TCLE). O cálculo amostral foi baseado no estudo Lichen e cols., de 2021, que descreveu uma diferença entre os grupos de pesquisa de 0,98 em escala Likert (3). Considerando um poder de 90%, com nível de significância de 5%, serão necessários 132 pacientes em cada grupo. Os participantes serão recrutados no PA do HSL, Unidade Bela Vista, São Paulo, Brasil, por assistente de pesquisa treinado. Este profissional estará de plantão por 25 horas semanais, distribuídas em cinco turnos de 5 horas cada entre 10:00 e 22:00, ao longo de 3 meses no período préintervenção e mais 3 meses no período pós-intervenção, totalizando 6 meses de recrutamento. O assistente poderá ser acionado pela equipe médica e/ou de enfermagem, além de realizar busca ativa por potenciais candidatos para o estudo. Os pacientes que forem considerados elegíveis serão convidados a assinar Termo de Consentimento Livre e Esclarecido (TCLE).

Entrevista inicial
A experiência do paciente e seu nível de conforto serão avaliados utilizando questionário desenvolvido com base nos estudos de Lichen e cols.
(2021) e McCusker e cols. (2019).
Também serão registrados os seguintes dados:
Nome completo, número de prontuário, número de atendimento, data de nascimento, CEP, sexo e data e horário de admissão no serviço de PA;
Fonte da informação, assim como, quando não for o próprio paciente, nome completo, data de nascimento, sexo e vínculo com o paciente;
Déficits sensoriais;
Histórico de quedas;
Risco de quedas segundo Memorial Emergency Department Fall Risk Assessment Tool (MEDFRAT) e Carpenter index (11, 12);
Comorbidade, fragilidade, severidade clínica, humor, vulnerabilidade geriátrica, alteração aguda do estado mental (delirium), experiência do
paciente, qualidade de vida, funcionalidade, e desempenho cognitivo, que serão avaliados através das escalas descritas a seguir.

Entrevista de seguimento

Para avaliação dos desfechos secundários, contaremos com um segundo assistente de pesquisa, que não terá acesso aos dados da avaliação inicial nem a informações sobre o atendimento realizado no PA, e conduzirá entrevista telefônica 30 dias após a avaliação inicial, com dois dias a mais ou a menos de tolerância. Neste contato serão avaliados revisitas hospitalares, ocorrência de quedas, incidência de delirium, qualidade de vida, humor, funcionalidade, desempenho cognitivo e ocorrência de óbito.

Entrevista com equipes médica e multidisciplinar

A entrevista com a equipe assistencial será realizada por assistente de pesquisa treinado e ocorrerá concomitante ao recrutamento e inclusão dos pacientes. Seguirá a padronização do estudo original de derivação e validação do carro de conforto (3) com escala de Likert para respostas às seguintes perguntas.

Revisão de prontuário e bancos de dados administrativos

Duração da estadia hospitalar e custos hospitalares serão extraídos do prontuário eletrônico do paciente e dos bancos de dados administrativos de inteligência do negócio do HSL (Business Intelligence, B.I.).

Todas as etapas do estudo serão desenvolvidas com o uso da ferramenta eletrônica de captura de dados Research Electronic Data Capture (REDCap) (67). O REDCap é um aplicativo seguro baseado na Web projetado para capturar informações, fornecendo: 1) uma interface intuitiva e validada para entrada de dados; 2) trilhas de auditoria para rastreamento de manipulação de dados e procedimentos de exportação; 3) procedimentos automatizados de exportação para downloads de dados contínuos para pacotes estatísticos comuns; e 4) procedimentos para importar dados de fontes externas (68). Os assistentes de pesquisa disporão de tablet com acesso online ao banco de dados do estudo para preenchimento direto, através do REDCap, da entrevista inicial, assim como para a aplicação e a assinatura do termo de consentimento livre e esclarecido.

Critério de Inclusão:

Atendimento no PA do HSL;

Idade igual ou maior a 65 anos;
Acomodação em box privativo, independentemente de indicação de internação. Capacidade de consentir e responder a entrevista ou presença de acompanhante habilitado a fazê-lo.

Critério de Exclusão:
Recusa em participar do estudo e/ou utilizar menu e carro de conforto;
Ausência de acompanhante capaz de consentir com a participação no estudo e fornecer as informações necessárias quando paciente com
alteração do estado mental ou comprometimento cognitivo;
Rebaixamento do nível de consciência;
Instabilidade hemodinâmica;
Insuficiência respiratória;
Impossibilidade de contato telefônico para entrevista.

Metodologia de Análise de Dados:
As análises serão realizadas com o pacote estatístico Stata versão 17 (StataCorp, College Station, TX). Todos os testes estatísticos serão bicaudais, admitindo-se um erro alfa de até 0,05. As variáveis numéricas serão reportadas como médias e desvios padrão, ou medianas e intervalos interquartis (IIQ) dependendo de sua distribuição. Esta será checada por inspeção visual dos histogramas de cada variável, com o cálculo dos coeficientes de assimetria (skewness) e curtose (kurtosis), e efetuação do teste de D¿Agostinho-Pearson para determinar a normalidade da distribuição, rejeitada com valores de p menores que 0,05. Algumas das variáveis também serão estratificadas em categorias para facilitar a interpretação clínica dos valores encontrados. Variáveis categóricas serão descritas em contagens absolutas e proporções. Comparações entre distribuições de variáveis numéricas utilizarão teste t de Student ou ANOVA se distribuição normal ou teste de Wilcoxon ou Kruskal-Wallis se distribuição não normal. As distribuições de variáveis categóricas serão comparadas utilizando teste do qui-quadrado, ou teste exato de Fisher quando apropriado.

Desfecho Primário:
Experiência do paciente.

Desfecho Secundário:

Experiência de profissionais das equipes médica e multidisciplinar;
Tempo de permanência hospitalar;
Custos hospitalares;
Revisitas hospitalares;
Quedas em 30 dias;
Incidência de delirium em 30 dias;
Qualidade de vida em 30 dias;
Funcionalidade em 30 dias;
Desempenho cognitivo em 30 dias.

Objetivo da Pesquisa:
Objetivo Primário:
Avaliar o impacto da implementação de menu e carro de conforto na experiência de pacientes idosos atendidos no PA.

Objetivo Secundário:
Avaliar o impacto da implementação de um menu de conforto e de um carro de conforto na experiência da equipe médica e multidisciplinar envolvida no cuidado de pacientes idosos atendidos no pronto-socorro.Avaliar o impacto da implementação de um menu de conforto e de um carrinho em outros desfechos exploratórios, incluindo duração da internação hospitalar, custos hospitalares e desfechos centrados no paciente até 30 dias em idosos atendidos no pronto-socorro, incluindo readmissões, quedas, incidência de delírium, qualidade de vida, humor, funcionalidade e desempenho cognitivo.

Avaliação dos Riscos e Benefícios:
Riscos:
A abordagem para entrada no estudo e entrevista inicial no serviço de PA tem como risco potencial a interferência na sua rotina de atendimento. Esse risco será minimizado garantindo a realização da entrevista em ambiente protegido e silencioso, com profissional técnico habilitado para coleta de informações e apenas após a implementação de todas as medidas definidas pela equipe médica. Também pode haver desconforto com o tempo dedicado pelo paciente e/ou por seu informante para responder as entrevistas inicial presencial e de acompanhamento telefônico, com estimativa de 15 minutos para a avaliação inicial e 10

minutos para entrevista de seguimento. Para minimizar esse risco, será garantido ao participante a escolha do momento que julgar mais adequado para a responder as entrevistas, assim como será garantido treinamento dos assistentes de pesquisa para que os tempos sejam minimizados dentro dos limites possíveis. Será assegurada ao participante a liberdade de recusar-se a participar da entrevista e de retirar-se do estudo a qualquer momento, caso seja a sua vontade, sem nenhum prejuízo ao seu atendimento ou seguimento no serviço, através da aplicação de TCLE. A quebra de sigilo será prevenida através do arquivo dos dados em plataforma com acesso restrito para processamento dos dados. Em caso de apresentações e colaboração por indivíduos externos, todos as identidades serão omitidas.

Benefícios:
Não haverá benefício direto aos participantes do estudo. Porém os resultados obtidos neste estudo poderão contribuir para a melhoria da qualidade do atendimento aos pacientes idosos em serviços de Pronto Atendimento (PA), avaliando intervenções inovadoras, como o menu e carro de conforto, para proporcionar maior bem-estar.

Comentários e Considerações sobre a Pesquisa:
Sem comentários adicionais sobre o projeto de pesquisa.

Considerações sobre os Termos de apresentação obrigatória:
Os documentos para os quais foram indicados pendência foram corrigidos adequadamente.

Recomendações:
Sem recomendações adicionais.

Conclusões ou Pendências e Lista de Inadequações:
Projeto cadastrado no CEPesq como HSL SP 2025-21, APROVADO nesta data conforme os documentos apresentados no pacote regulatório.

Lembramos que, conforme o item XI.2.d da Res. 466/2012 o pesquisador deverá manter o CEPesq informado sobre o andamento de sua pesquisa através do envio de relatórios parciais (semestrais) e final. De acordo com o cronograma apresentado, está previsto a entrega do relatório parcial em 06 meses após a data da aprovação. Caso haja alterações no cronograma do estudo, por favor, submeter atualização.

Considerações Finais a critério do CEP:

Este parecer foi elaborado baseado nos documentos abaixo relacionados:

Tipo Documento	Arquivo	Postagem	Autor	Situação
Informações Básicas do Projeto	PB_INFORMAÇÕES_BÁSICAS_DO_PROJETO_2486051.pdf	06/02/2025 11:47:11		Aceito
Folha de Rosto	Folha_de_Rosto.pdf	06/02/2025 11:46:44	Pedro Kallas Curiati	Aceito
Declaração de Pesquisadores	Carta_Resposta_CEP_HSL.doc	06/02/2025 11:46:11	Pedro Kallas Curiati	Aceito
TCLE / Termos de Assentimento / Justificativa de Ausência	TCLE_equipe.docx	06/02/2025 11:45:41	Pedro Kallas Curiati	Aceito
TCLE / Termos de Assentimento / Justificativa de Ausência	TCLE_paciente.docx	06/02/2025 11:45:26	Pedro Kallas Curiati	Aceito
Projeto Detalhado / Brochura Investigador	Projeto.docx	06/02/2025 11:44:42	Pedro Kallas Curiati	Aceito
Declaração de concordância	AUTORIZACAO_AREA.pdf	21/01/2025 16:12:42	MARIANE TAMI AMANO	Aceito
Declaração de Pesquisadores	TERMO_COMPROMISSO_PESQUISADOR.pdf	21/01/2025 16:12:27	MARIANE TAMI AMANO	Aceito

Situação do Parecer:
Aprovado

Necessita Apreciação da CONEP:
Não

SAO PAULO, 12 de Março de 2025

Assinado por:
Mirian de Freitas Dal Ben Corradi
(Coordenador(a))
