# Supplementary material for: Enhancing the emergency department experience for older adults: Study protocol for the implementation of a comfort menu and cart
Source: PLoS One. 2025 Dec 4;20(12):e0332773. doi: 10.1371/journal.pone.0332773 (PMC12677483; doi:10.1371/journal.pone.0332773)
Supplement: S2 File — (PDF) [file pone.0332773.s003.pdf]

Machine Translated by Google

SYRIAN-LEBANESE HOSPITAL / CHARITABLE SOCIETY OF LADIES

SUBSTANTIATE OPINION OF THE CEP

RESEARCH PROJECT DATA

Research Title: Non-pharmacological interventions to improve the elderly patient experience in Emergency care: implementation of menu and comfort car in a tertiary philanthropic hospital

Researcher: Pedro Kallas Curiati

Thematic Area:

Version: 2

CAAE: 85870125.4.0000.5461

Proposing Institution: Ladies' Charitable Society of the Syrian-Lebanese Hospital

Main Sponsor: Ladies' Charitable Society of the Syrian-Lebanese Hospital

OPINION DATA

Opinion Number: 7,436,261

Project Presentation:

Design:

This study will use a quasi-experimental, before-and-after design.

Summary:

INTRODUCTION: Population aging is a global trend, with projections indicating a significant increase in the proportion of individuals aged 65 and older by 2050. This scenario imposes the need to adapt emergency department (ER) services to meet the specific demands of older patients, who often have multiple comorbidities and face challenges such as sensory and cognitive difficulties. International guidelines, such as those published by the American College of Emergency Physicians and the European Task Force on Geriatric Emergency Medicine, recommend modifications to the physical environment and staff training to improve care in geriatric ERs.

In a recent study published in 2021, Lichen et al. demonstrated that non-pharmacological interventions, such as a "comfort car," improve the experience of elderly patients in the ER. OBJECTIVES: To evaluate the impact of implementing a menu and a "comfort car" on the experience of elderly patients treated in the ER, the experience of professionals from the medical and multidisciplinary teams involved in the care of elderly patients in the ER, and the length of stay.

Machine Translated by Google

hospital stay, hospital costs, and other patient-centered outcomes within 30 days, including hospital revisits, falls, incidence of delirium, quality of life, functional status, and cognitive performance.
METHODS: A before-and-after study will be conducted to recruit patients over 65 years of age treated in the private emergency rooms of Hospital Sírio Libanês (HSL). Inclusion criteria will be: treatment in the HSL emergency room; age 65 or older; accommodation in a private room, regardless of indication for hospitalization; and ability to consent and respond to the interview or presence of a companion able to do so. Exclusion criteria will include: refusal to participate in the study and/or use the menu and comfort car; absence of a companion able to consent to participation in the study and provide the necessary information when the patient presents with altered mental status or cognitive impairment; decreased level of consciousness; hemodynamic instability; acute respiratory failure; and inability to be contacted by telephone for interview.

At least 132 patients will be included in each phase of the study (pre- and post-comfort car implementation). Patients will be initially interviewed to obtain demographic, clinical, and ED experience data. In the post-comfort car implementation phase, questions regarding their perception of its impact on experience and comfort during the ED stay will be added. A follow-up telephone interview will be conducted 30 days after enrollment to assess clinical outcomes of interest, including hospital revisits, falls, incidence of delirium, quality of life, mood, functionality, and cognitive performance. Analyses will be performed with Stata 17, using two-tailed statistical tests with an alpha error of 0.05. Numerical variables will be presented as means and standard deviations or medians and interquartile ranges, depending on the distribution. Categorical variables will be described as counts and proportions. Comparisons of numerical variables will be made with Student's t-test or ANOVA for normal distributions and the Wilcoxon or Kruskal-Wallis test for non-normal distributions.

Categorical variables will be compared using the chi-square test or Fisher's exact test, if necessary.
EXPECTED RESULTS: We hope to demonstrate the impact of implementing menu and cart comfort in PA in all studied outcomes.

Introduction:
Population aging is an undeniable reality both in Brazil and globally (1). According to the 2019 report by the Department of Economic and Social Affairs of the United Nations (UN), the global population aged 65 and over

Machine Translated by Google

will increase from 9.1% to 11.7% in 2030 and 15.9% in 2050, surpassing the number of young people aged 15 to 24 (1). The number of people over 80 years of age is also growing significantly worldwide, with a projected 426 million individuals in this age group by 2050 (1). Elderly patients tend to have multiple comorbidities, which leads them to seek emergency care (ER) services more frequently (2, 3). However, these services are often not adapted to adequately address their particular needs (2). Visual and hearing difficulties, decline in functional reserve, cognitive impairment due to delirium and/or dementia, and impaired communication are common problems that can be exacerbated by the stressful ER environment (2, 4). To address these challenges, specific care guidelines have been developed by international organizations. The American College of Emergency Physicians, in conjunction with the American Geriatrics Society, the Emergency Nurses Association, and the Society for Academic Emergency Medicine, published the "Geriatric Emergency Department Guidelines" in 2014 (4). These guidelines contain detailed recommendations on modifying the physical environment, training staff, and implementing care processes specific to older patients (4). These include creating more comfortable waiting areas, using adequate lighting, reducing noise, and providing walking aids (4). The European Task Force on Geriatric Emergency Medicine published recommendations for the care of older adults in the emergency department in 2020, emphasizing the importance of comprehensive assessment, including screening for common conditions in older adults, such as delirium, depression, and risk of falls (5). This document also suggests implementing specific protocols for the management of chronic and acute conditions, as well as integrating palliative care when appropriate (5). A common recommendation across both guidelines is ongoing training of healthcare staff in geriatric competencies, including effective communication with older patients and identification and management of geriatric syndromes (4, 5). Designating "geriatric champions" or geriatric resource persons within emergency departments aims to ensure appropriate care based on best practices (4, 5). A study conducted in an emergency department in the United States and published by Lichen et al. in 2021 explored the impact of nonpharmacological interventions on improving the comfort and experience of older patients (3). This study introduced the concept of a "comfort cart," a mobile cart containing low-cost items such as reading glasses,

hearing aids, heated blankets, and entertainment materials such as large-print books and magazines, with the aim of improving communication between patients

Machine Translated by Google

elderly patients and healthcare staff, in addition to providing a more pleasant experience during waiting and care (3). The "comfort cart" was made available in waiting areas and inside the treatment rooms, allowing patients to access these items as needed (3). Furthermore, nursing staff received specific training to use these resources effectively and to identify the individual needs of elderly patients. The impact of this intervention was assessed through satisfaction questionnaires completed by patients and healthcare staff before and after the implementation of the comfort cart, with very positive results (3). It was concluded that simple, low-cost, humanized, and patient-centered interventions can have a substantial impact on the experience of patients and their professionals in the ED (3). To the best of our knowledge, there are no other studies on the use of comfort carts for elderly patients in ED settings. Stolzman et al., in 2020, explored a similar intervention in the intensive care unit (ICU), led by nurses, to improve care for critically ill patients and their families (6). The initiative included welcoming items and emphasized the importance of an environment that promotes well-being, especially in end-of-life situations (6). This study also revealed the strengthening of teamwork and patient-centered care, with improved ICU experience and attention to families' emotional needs (6). McCusker et al., in 2018, explored the experience of elderly patients in emergency care and proposed measures for their assessment (2). They highlighted the specific challenges these patients face and indicated that their needs may differ from those of younger patients (2). The authors created and validated two scales to assess relevant aspects of emergency care for older adults: one focused on personal care and communication and the other on waiting time. Both were significantly associated with the perception of care quality and the propensity to return to the same emergency care service (2). Their application was proposed to improve the experience of older adults by adapting care to their specific needs and promoting a more welcoming and responsive environment for this group (2).

Hypothesis:

The implementation of a menu and a "comfort cart" in the ER of Hospital Sírio-Libanês (HSL) will improve the experience of patients and care teams, as well as length of hospital stay, hospital costs and other patient-centered outcomes within 30 days, including hospital revisits, falls, incidence of delirium, quality of life,

Machine Translated by Google

functionality, and cognitive performance.

Proposed Methodology: All
participants will be asked to sign an informed consent form (ICF). The sample size calculation was based on the 2021 study by Lichen et al., which reported a difference between the research groups of 0.98 on a Likert scale (3). Considering a power of 90%, with a significance level of 5%, 132 patients will be required in each group. Participants will be recruited at the HSL Emergency Room, Bela Vista Unit, São Paulo, Brazil, by a trained research assistant. This professional will be on call for 25 hours per week, distributed in five 5-hour shifts between 10:00 AM and 10:00 PM, over a 3-month pre-intervention period and an additional 3 months post-intervention period, totaling 6 months of recruitment. The assistant may be contacted by the medical and/or nursing staff, in addition to actively searching for potential candidates for the study. Patients who are considered eligible will be invited to sign an Informed Consent Form (ICF).

Initial interview
The patient's experience and comfort level will be assessed using a questionnaire developed with based on studies by Lichen et al. (2021) and McCusker et al. (2019).
The following data will also be recorded: Full name, medical record number, service number, date of birth, zip code, gender and date and time of admission to the ER service; Source of information, as well as, when it is not the patient himself, full name, date of birth, gender and relationship with the patient;

Sensory deficits;
History of falls; Risk of falls according to the Memorial Emergency Department Fall Risk Assessment Tool (MEDFRAT) and Carpenter index (11, 12); Comorbidity, frailty, clinical severity, mood, geriatric vulnerability, acute change in mental status (delirium), patient experience, quality of life, functionality, and cognitive performance, which will be assessed using the scales described below.

Machine Translated by Google

Follow-up Interview: To
assess secondary outcomes, we will have a second research assistant, who will not have access to the initial assessment data or information about the care provided in the ER. The assistant will conduct a telephone interview 30 days after the initial assessment, plus or minus two days. This interview will assess hospital revisits, occurrence of falls, incidence of delirium, quality of life, mood, functionality, cognitive performance, and occurrence of death.

Interview with medical and multidisciplinary teams.
The interview with the healthcare team will be conducted by a trained research assistant and will occur concurrently with patient recruitment and inclusion. It will follow the standardization of the original study for derivation and validation of the comfort car (3), with Likert-based responses to the following questions.

Review of medical records and administrative databases
Length of hospital stay and hospital costs will be extracted from the patient's electronic medical record and HSL's administrative business intelligence (BI) databases.

All stages of the study will be developed using the Research Electronic Data Capture (REDCap) electronic data capture tool (67). REDCap is a secure web-based application designed to capture information, providing: 1) an intuitive and validated interface for data entry; 2) audit trails for tracking data manipulation and export procedures; 3) automated export procedures for continuous data downloads to common statistical packages; and 4) procedures for importing data from external sources (68). Research assistants will have a tablet with online access to the study database to directly complete the initial interview through REDCap, as well as to administer and sign the consent form.

free and informed consent.

Inclusion Criteria:

Service at the HSL Emergency Room;

Machine Translated by Google

Age 65 or older; Accommodation in a
private box, regardless of indication for hospitalization. Ability to consent and respond to an interview or presence of a companion authorized to do so.

Exclusion Criteria:

Refusal to participate in the study and/or use the menu and comfort car;
Absence of a companion capable of consenting to participation in the study and providing the necessary information when the patient has

altered mental status or cognitive impairment;
Lowering of the level of consciousness;
Hemodynamic instability;
Respiratory failure;
Impossibility of telephone contact for interview.

Data Analysis Methodology: Analyses will
be performed using the Stata statistical package version 17 (StataCorp, College Station, TX).
All statistical tests will be two-tailed, allowing for an alpha error of up to 0.05. Numerical variables
will be reported as means and standard deviations, or medians and interquartile ranges (IQR) depending on their distribution. This will be checked by visual inspection of the histograms of each variable, with the calculation of the coefficients of asymmetry (skewness) and kurtosis (kurtosis), and performing the D'Agostinho-Pearson test to determine the normality of the distribution, rejected with p-values less than 0.05.

Some of the variables will also be stratified into categories to facilitate clinical interpretation of the values found. Categorical variables will be described as absolute counts and proportions.
Comparisons between distributions of numerical variables will use Student's t-test or ANOVA if the distribution is normal, or the Wilcoxon or Kruskal-Wallis test if the distribution is non-normal. Distributions of categorical variables will be compared using the chi-square test or Fisher's exact test when appropriate.

Primary Outcome:

Patient experience.

Secondary Outcome:

Machine Translated by Google

Experience of professionals from medical and multidisciplinary teams;
Length of hospital stay;
Hospital costs;
Hospital visits;
Falls in 30 days;
Incidence of delirium in 30 days;
Quality of life in 30 days;
Functionality in 30 days;
Cognitive performance in 30 days.

Research Objective:

Primary Objective:

Evaluate the impact of implementing a menu and comfort cart on the experience of elderly patients attended at the PA.

Secondary Objective:

To evaluate the impact of implementing a comfort menu and a comfort cart on the experience of the medical and multidisciplinary team involved in the care of elderly patients treated in the emergency department. To evaluate the impact of implementing a comfort menu and a cart on other exploratory outcomes, including length of hospital stay, hospital costs, and patient-centered outcomes up to 30 days in elderly patients treated in the emergency department, including readmissions, falls, incidence of delirium, quality of life, mood, functionality, and cognitive performance.

Risk and Benefit Assessment:

Risks:

The approach to study entry and the initial interview at the ER service carries the potential risk of interfering with your routine care. This risk will be minimized by ensuring the interview is conducted in a protected and quiet environment, with a qualified technical professional to collect information, and only after all measures defined by the medical team have been implemented. There may also be discomfort with the time dedicated by the patient and/or their informant to respond to the initial in-person and telephone follow-up interviews, with an estimated 15 minutes for the initial assessment and 10

Machine Translated by Google

minutes for the follow-up interview. To minimize this risk, participants will be given the opportunity to choose the most appropriate time to complete the interviews, and research assistants will be trained to minimize interview time as much as possible. Participants will be guaranteed the freedom to refuse to participate in the interview and to withdraw from the study at any time, if they so desire, without any detriment to their care or follow-up, through the application of a free and informed consent form. Breach of confidentiality will be prevented by archiving the data on a platform with restricted access for data processing. In the event of presentations and collaboration by external individuals, all identities will be concealed.

Benefits:

There will be no direct benefit to study participants. However, the results obtained in this study may contribute to improving the quality of care for elderly patients in emergency departments.
Service (PA), evaluating innovative interventions, such as the menu and comfort car, to provide
greater well-being.

Comments and Considerations on the Research:
No additional comments on the research project.

Considerations on the Mandatory Submission Terms: The
documents for which pending submissions were indicated have been appropriately corrected.

Recommendations:
No additional recommendations.

Conclusions or Pending Issues and List of
Inadequacies: Project registered with CEPesq as HSL SP 2025-21, APPROVED on this date in accordance with the documents presented in the regulatory package.

We remind you that, according to item XI.2.d of Res. 466/2012, the researcher must keep CEPesq informed about the progress of his/her research by sending partial (half-yearly) and final reports.
According to the presented schedule, the partial report is expected to be delivered within six months of the approval date. If there are any changes to the study schedule, please submit an update.

Machine Translated by Google

Final Considerations at the discretion of the CEP:

This opinion was prepared based on the documents listed below:

Document Type	File	Posted	Author	Situation
Basic Information of the Project	PB_BASIC_INFORMATION_OF_P PROJECT_2486051.pdf	02/06/2025 11:47:11		Accepted
Page	Cover Page.pdf Cover	06/02/2025 11:46:44	Pedro Kallas Curiati	Accepted
Declaration of Researchers	Reply_Letter_CEP_HSL.doc	06/02/2025 11:46:11	Pedro Kallas Curiati	Accepted
TCLE / Terms of Assent / Justification of Absence	TCLE_equipe.docx	06/02/2025 11:45:41	Pedro Kallas Curiati	Accepted
TCLE / Terms of Assent / Justification of Absence	TCLE_patient.docx	06/02/2025 11:45:26	Pedro Kallas Curiati	Accepted
Detailed Design / Brochure Researcher	Project.docx	06/02/2025 11:44:42	Pedro Kallas Curiati	Accepted
Declaration of agreement	AUTHORIZATION_AREA.pdf	January 21, 2025 16:12:42	MARIANE TAMI AMANO	Accepted
Declaration of Researchers	TERM_COMMITMENT_RESEARCH DOR.pdf	January 21, 2025 16:12:27	MARIANE TAMI AMANO	Accepted

Opinion Status:
Approved

Needs CONEP's Appreciation:
No

SAO PAULO, March 12, 2025

Signed by:
Mirian de Freitas Dal Ben Corradi
(Coordinator)
