# Supplementary material for: Enhancing the emergency department experience for older adults: Study protocol for the implementation of a comfort menu and cart
Source: PLoS One. 2025 Dec 4;20(12):e0332773. doi: 10.1371/journal.pone.0332773 (PMC12677483; doi:10.1371/journal.pone.0332773)
Supplement: Attachment — Submitted filename: Response to reviewers.docx [file pone.0332773.s005.docx]

**Response to Reviewers' Comments**

We thank the reviewer for the insightful feedback and constructive suggestions, which have greatly helped us improve the quality of our manuscript. Below, we provide a point-by-point response to each comment, detailing how the revisions have been incorporated into the updated submission.

**Reviewer #1**

**Comment:** "*The only critique/comment I have about the study design pertains to the timing of the roll-out of the cart. The study plans to enroll the pre group over a period of 3 months (which seems appropriate based on feasibility numbers and power calculations). The study then plans to immediately enroll the batch of post participants. This leaves no time for roll-out/equipping of the cart and training in proper use of the cart. It also doesn't allow for any hiccups in the cart roll-out process. This reviewer would suggest including a roll-out phase for the care cart of a month before enrolling participants in a study which will use the cart. This will allow for any potential problems to be resolved before participants are enrolled.*"

**Response:** We thank the reviewer for this crucial suggestion. We fully agree that a dedicated rollout period is essential to ensure the seamless implementation of the comfort menu and cart, allowing for proper staff training and resolution of potential logistical challenges prior to the post-intervention data collection. We have incorporated this recommendation into the "Intervention" section of the manuscript (page 17, lines 13-21) to reflect this important preparatory phase:

"A one-month dedicated rollout period will precede the commencement of post-intervention participant enrollment. This phase is designed for comprehensive staff training on the proper use and components of the comfort menu and cart, including familiarization with all available items and menu options. Additionally, this period will ensure all necessary equipment is adequately prepared and any potential logistical challenges associated with the cart's implementation are identified and resolved, thereby optimizing the fidelity and effectiveness of the intervention before data collection for the post-intervention group begins."
